# DDPM: A Dengue Disease Prediction and Diagnosis Model Using Sentiment Analysis and Machine Learning Algorithms

**DOI:** 10.3390/diagnostics13061093

**Published:** 2023-03-14

**Authors:** Gaurav Gupta, Shakir Khan, Vandana Guleria, Abrar Almjally, Bayan Ibrahimm Alabduallah, Tamanna Siddiqui, Bader M. Albahlal, Saad Abdullah Alajlan, Mashael AL-subaie

**Affiliations:** 1 Yogananda School of AI, Computers and Data Sciences, Shoolini University, Solan 173229, India; 2 College of Computer and Information Sciences, Imam Mohammad Ibn Saud Islamic University (IMSIU), Riyadh 11564, Saudi Arabia; 3 Department of Computer Science and Engineering, University Centre for Research and Development, Chandigarh University, Mohali 140413, India; 4 School of Bioengineering & Food Technology, Shoolini University, Solan 173229, India; 5 Department of Information System, College of Computer and Information Sciences, Princess Nourah bint Abdulrahman University, Riyadh 11564, Saudi Arabia; 6 Department of Computer Science, Aligarh Muslim University, Aligarh 202001, India

**Keywords:** dengue fever, machine learning, prediction, classification, SVM, decision tree, random forest, ANN, GNB, opinion mining, sentiment analysis

## Abstract

The aedes mosquito-borne dengue viruses cause dengue fever, an arboviral disease (DENVs). In 2019, the World Health Organization forecasts a yearly occurrence of infections from 100 million to 400 million, the maximum number of dengue cases ever testified worldwide, prompting WHO to label the virus one of the world’s top ten public health risks. Dengue hemorrhagic fever can progress into dengue shock syndrome, which can be fatal. Dengue hemorrhagic fever can also advance into dengue shock syndrome. To provide accessible and timely supportive care and therapy, it is necessary to have indispensable practical instruments that accurately differentiate Dengue and its subcategories in the early stages of illness development. Dengue fever can be predicted in advance, saving one’s life by warning them to seek proper diagnosis and treatment. Predicting infectious diseases such as dengue is difficult, and most forecast systems are still in their primary stages. In developing dengue predictive models, data from microarrays and RNA-Seq have been used significantly. Bayesian inferences and support vector machine algorithms are two examples of statistical methods that can mine opinions and analyze sentiment from text. In general, these methods are not very strong semantically, and they only work effectively when the text passage inputs are at the level of the page or the paragraph; they are poor miners of sentiment at the level of the sentence or the phrase. In this research, we propose to construct a machine learning method to forecast dengue fever.

## 1. Introduction

The Aedes aegypti mosquito handles transmitting the DENV virus, the causative agent of dengue fever, from person to person. There is currently no vaccination that can protect against all virus serologies. This is because there is no such thing as a vaccine. As a direct consequence, trying to reduce the number of mosquitoes in an area has become the primary focus of the fight against the disease. Researchers are using machine learning (ML) and deep learning (DL) to forecast dengue cases and assist governments in their fight against the disease [1].

Dengue virus is a flavivirus, a genus of flaviviruses, and a family of Flaviviridae [2,3]. Arthropods are the primary vectors for the spread of the dengue virus. It can be broken down into four serotypes, referred to by the names DEN 1, DEN 2, DEN 3, and DEN 4. According to the World Health Organization (WHO), dengue fever poses a considerable risk to the public health of countries all over the world, especially those nations that are in tropical or subtropical regions (WHO). There are approximately 2.5 billion people who live in dengue-endemic areas [4], with an annual infection rate of 400 million individuals and a mortality rate that can vary from 5 to 20 percent in specific locations [5]. Dengue fever is a disease that may be found all over the world. However, it is particularly prevalent in some regions, such as Europe and the United States of America (USA) [6]. The first recorded case of dengue fever in India occurred in Madras in the year 1780, and the first pandemic of dengue disease in India was confirmed by virological testing in the years 1963–1964 [7].

Dengue fever is a dangerous illness that manifests similarly to the flu and can afflict persons of any age, including infants, children, adolescents, and adults [8]. The Aedes aegypti mosquito is to blame for the transmission of the disease to humans, which takes place more often during the wetter months [9]. The World Health Organization (WHO) distinguishes between two levels of severity for dengue fever: moderate and severe [10,11]. Extreme cases are characterized by an abnormal amount of bleeding, impairment of organ function, or significant loss of plasma, while others are considered to be relatively uncomplicated [11]. According to the categorization used in 1997, dengue fever can be broken down into three subtypes: undifferentiated fever, D.F., and DHF [12]. The DHF served as the basis for the creation of grades I–IV. D.F. can develop because of primary diseases as well as secondary diseases, and it is most common in adults and children who have developed into adults. The onset of symptoms is typically accompanied by a high temperature that cycles between phases and can continue anywhere from one week to three weeks [13]. A metallic taste, loss of appetite, diarrhea, nausea, and stomachache are some of the other symptoms. Other symptoms include severe headaches, particularly retrobulbar migraines, fatigue, myalgia, and painful joints. Dengue fever is also referred to as break-bone fever [10,14] because the condition is often accompanied by myalgia and discomfort in the joints.

The worldwide public health system has a continuing need for the early detection of dengue fever, and machine learning algorithms may help medical professionals recognize and prevent infection at an earlier stage. This would save time, money, and the uncomfortable experience of pathology testing [15,16]. Diagnostics would benefit significantly from this. In medical diagnostics, machine learning algorithms have been used to diagnose conditions based on clinical and laboratory signs and produce the outcome, as stated in [17]. This has been carried out so that the outcome will be as accurate as possible. They also note that artificial neural networks (ANN) are one of the most prominent ways of addressing medical diagnostic issues and that support vector machines (SVM) give correct conclusions when evaluating a single ailment. Both concepts are discussed in the article. These two assertions are contained within the same piece of writing altogether.

In another publication [18], the authors use an artificial neural network to assess meteorological data obtained from Singapore’s National Environment Agency (SNEA). To make a prediction about dengue illness in Thailand, [19] employed meteorological data collection and implemented feature selection techniques. SVM is another well-known method that can be used to address this matter. Utilizing support vector machines (SVM) on a Singapore meteorological dataset can help with the prediction of dengue fever [20,21]. On a small sample from Brazil, Ref. [22] uses gene expression data and an RBF kernel. Authors of [23] also use the support vector regression (SVR) method. They use data from the Guangdong region to compare several machine learning algorithms (China). Ref. [24] use climatic factors to investigate the incidence of dengue fever in the Philippines. They compare random forest, gradient boosting, general additive modeling, and seasonal autoregressive integrated moving average with exogenous variables [25,26]. These days, deep learning strategies are getting a lot of attention as a potential solution to a variety of challenges, particularly in the discipline of medical imaging. Some of the features that will be included in this system are the ability to suggest differential diagnoses, the composition of preliminary radiology reports, automatic detection, and quantitative characteristics of the lesion in medical imaging. However, this does not imply that the replacement of radiologists is dangerous; instead, it helps physicians provide more accurate diagnoses to their patients. The subfield of computer vision, known as deep learning, is considered an advanced subfield. The primary aim of computer vision is to carry out a variety of tasks simultaneously, including picture detection and recognition, image analysis, natural language processing, and other similar activities. Over the course of the past few years, interest in computer vision has grown substantially across a variety of academic domains. CNN is used in most computer vision tasks, particularly those involving the classification, recognition, and segmentation of medical pictures. The convolutional neural network (CNN) is a sort of artificial neural network that was developed specifically for processing data related to images and videos. It begins with photographs as input, then extracts and learns features from those images, and then classifies output images depending on the features it has learned from the input images. There have been several different CNN-based model ideas put forward, including AlexNet, SPP-Net, VGGNet, ResNet, GoogleLeNet, and others. Deep convolutional neural network (CNN)-based algorithms have shown promising results in the processing of medical pictures. An introduction to CNN in medical imaging analysis as well as a general discussion of machine learning and deep learning applied to medical pictures are included in this work. The researchers investigate several different machine learning methods, and general additive modeling is just one of them.

The contribution of work:

The purpose of this paper is to pursue an early diagnostic model that helps doctors in the prompt prognosis and diagnosis of dengue disease by using machine learning algorithms. The key steps are as follows:

1. Using techniques from the field of machine learning, such as the KNN classifier, decision tree, random forest, Gaussian naive Bayes, and support vector classifier (SVC), among others.
2. Creating a diagnostic model based on machine learning for fast detection and prognosis of dengue disease to aid medical professionals in making decisions.
3. The K-Fold method is used here for the purpose of result validation.

## 2. Related Work

The field of computing known as machine learning (ML) enables computers to access information without the requirement for any encoding [27,28]. The study of ML falls under the umbrella of the discipline of computer science. MML has become all-pervasive and essential for resolving intricate problems in any science department, but especially in the field of illness diagnostics [29,30]. Machine learning algorithms and techniques will soon be able to foresee and differentiate between a wide variety of illnesses in the healthcare field [30,31,32]. This is a direct effect of ongoing technological improvement. Machine learning is often cited as one of the most productive research approaches, mainly when predicting disease occurrence. There are several distinct kinds of ML algorithms, each of which is capable of being applied for the purpose of disease forecasting [33,34]. The findings of an investigation into several different machine learning algorithm approaches are shown in Table 1, along with the research that is pertinent to the topic. According to the findings of the review that was carried out [35,36], several distinct machine learning methods, including SVM, KNN, R.F., D.T., and SVC, are utilized and evaluated for the purpose of dengue prediction.

## 3. Materials and Methods

**Figure 1:**
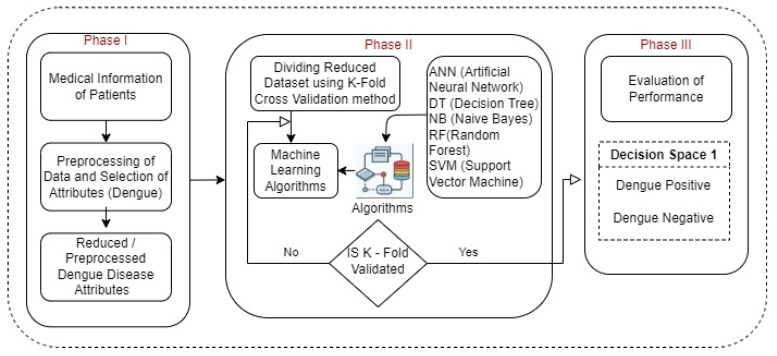
The workflow for the implementation of the proposed diagnostic model for the diagnosis of dengue disease.

Within the scope of this publication, we constructed a diagnostic and prognostic model for dengue fever. We broke the task down into steps, beginning with the first phase of data collection, then moving on to data preprocessing, and finally employing ML classifiers to evaluate the output according to the accuracy (mean) of disease prediction (see Figure 1).

### 3.1. Data Collection

The objective is to accurately predict the total number of dengue cases present in the test set, which will be labeled against each city, year, and week of the year. This study uses data from the DengAI competition (open data of dengue illness competition: DengAI: Predicting Disease Spread (drivendata.org)). The DengAI competition comprises data for two cities, San Juan and Iquitos, extending from three to five years. Every piece of information contributes its own set of forecasts for these cities. The data are separated into two categories: the training and test datasets, as shown in Table 2.

### 3.2. Data Preprocessing

**Figure 2:**
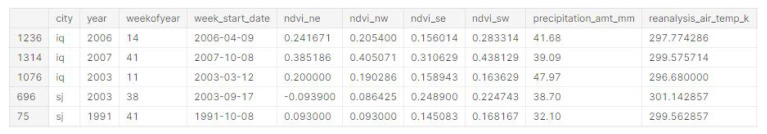
Sample data were taken for the study.

The machine learning pipeline’s most significant component is the step known as “data preprocessing.” Data preprocessing converts unprocessed data into processed (meaningful) data. The dataset needs to be cleaned, normalized, and completely free of noise before it can be used for analysis (see Figure 2).

### 3.3. Features Selection

In building a prediction model, one of the most critical steps is called “feature selection.” During this phase, the number of variables (or inputs) is narrowed down to reduce the amount of computing required for the modeling process and, in some cases, to improve the overall performance of the model. The dataset has missing data for certain of its attributes, so we use the mean method to replace those values. After that, we use the fit and transform method to normalize and standardize the data.

**Figure 3:**
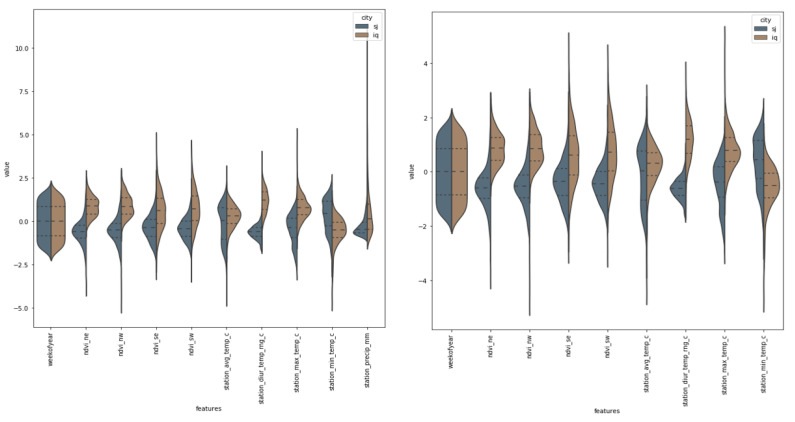
The dengue dataset utilizes a variety of features versus values for its parameters.

We can see that there are several different features that have extreme values by looking at Figure 3. After investigating the data, it became clear that they are neither outliers nor errors; hence, we are unable to disregard them and will have to take them into consideration. The values of precipitation are taken into consideration here, and given that these are estimates of the amount of rain, it is reasonable to anticipate that the weather can vary significantly depending on the location.

The features reanalysis_avg_temp_k and reanalysis_specific_humidity_g_per_kg appear to be pretty similar in shape; nonetheless, the question that arises here is whether or not they are correlated with one another.

**Figure 4:**
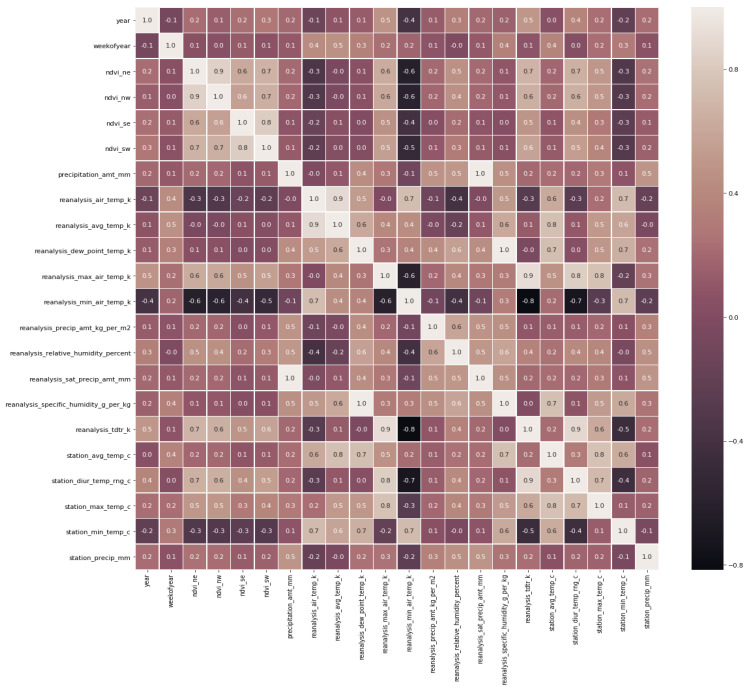
Correlation among features.

By looking at Figure 4, we can come to the conclusion that certain features are perfectly associated with one another (1), while other features are practically perfectly correlated with one another (0.9). The same information is presented in Table 3.

As we want to detect dengue in this manuscript for the same, if features are far in two cities, then it is suitable for ML classification (reanalysis_tdtr_k); otherwise, if they are near and give mixed information about features, then it is not considered suitable for prediction/classification.

**Figure 5:**
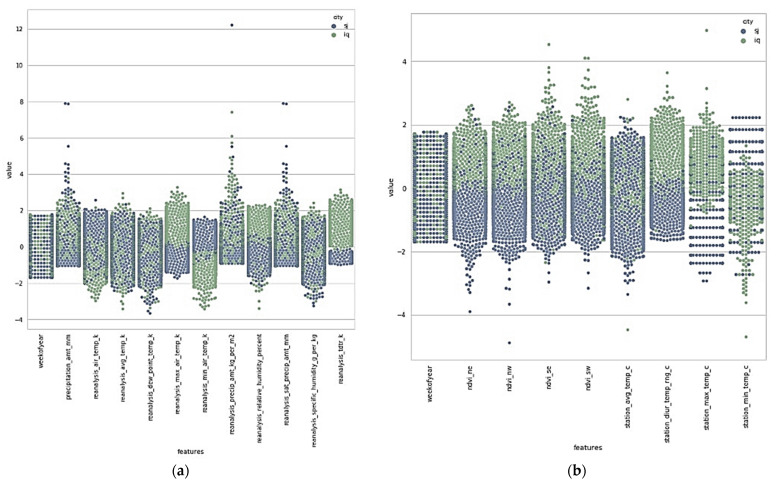
Various parameters were used in the dengue dataset, along with their characteristics. (**a**) Values along with Features. (**b**) Various parameters were used in the dengue dataset, along with their characteristics.

**Figure 6:**
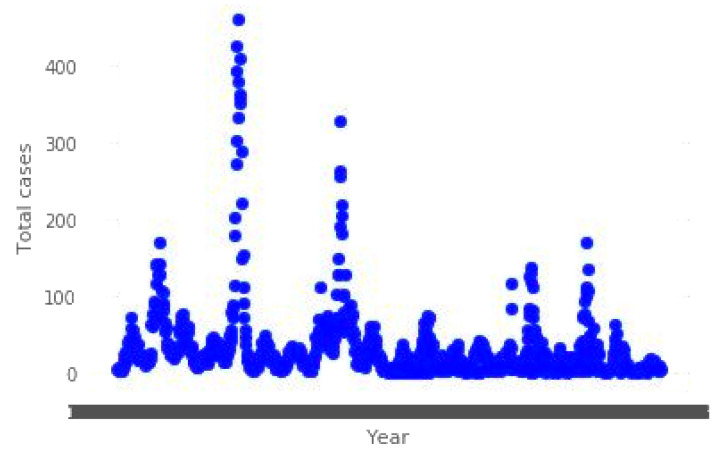
Total cases in the year.

From Figure 5 and Figure 6, and from the dataset, we can create a new data frame, i.e., X_train plus the total_cases column of y_train.

After applying all the above-mentioned steps, we deduce the features from the dataset, as shown in Table 4.

In this feature selection, we are dropping out the two features, i.e., reanalysis_sat_precip_amt_mm and reanalysis_specific_humidity_g_per_kg. At this time, we are not considering them because they are almost perfectly correlated (0.9), and we want to achieve good accuracy. However, with the present scenario, if we go for machine learning algorithms, i.e., KNN, D.T., R.F., and GNB, the accuracy comes out to be significantly less. This is due to the total number of cases immensely varying from 0 to 400+. The question arises, “How can we improve this accuracy?” The answer to this question is to divide our dataset into two cities, as shown in Table 4.

**Figure 7:**
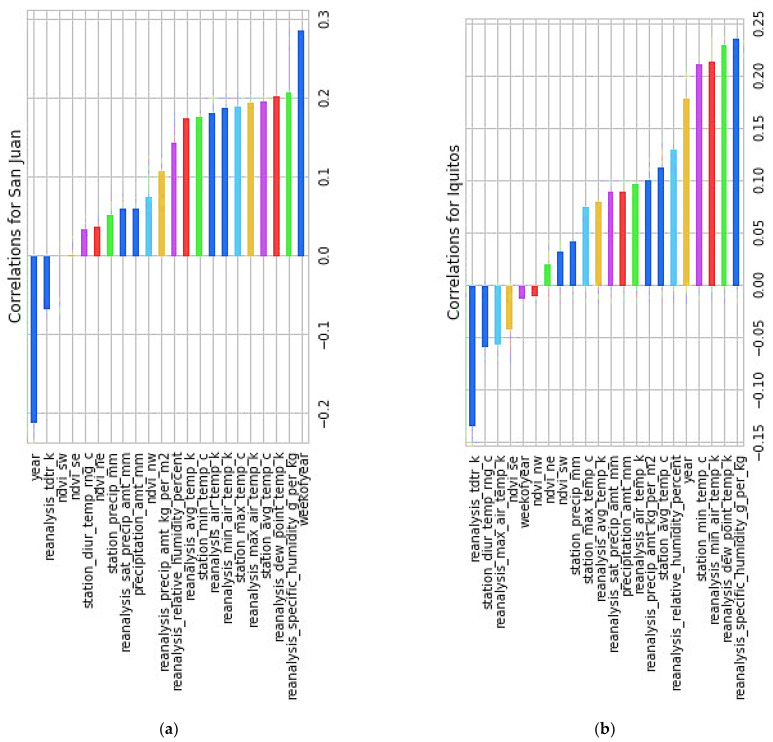
Correlation for two cities, San Juan and Iquitos. (**a**) Correlation in San Juan. (**b**) Correlation in Iquitos.

After this, we will find the correlation for two different cities, i.e., San Juan and Iquitos, separately, shown in Figure 7.

After finding the correlation between the two cities, we can deduce some information, such as that both cities showed promising results for

reanalysis_specific_humidity_g_per_kg
reanalysis_dew_point_temp_k
reanalysis_min_air_temp_k

**Figure 8:**
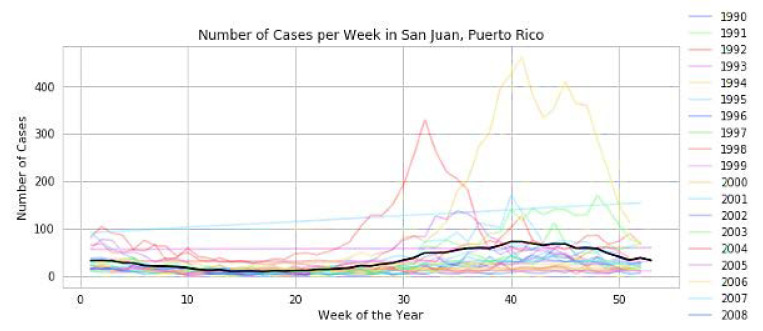
Increase in outbreaks and cases in San Juan city of Puerto Rico in weeks.

**Figure 9:**
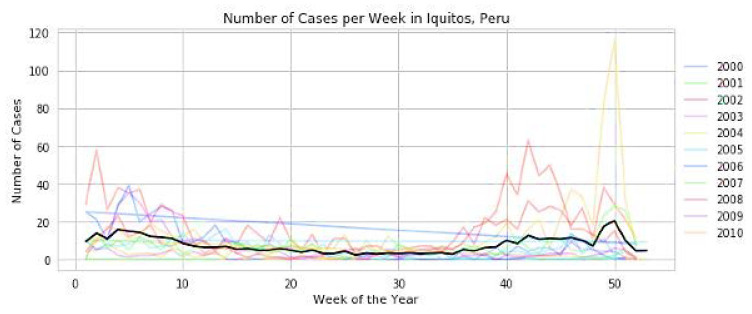
Increase in outbreaks and cases in Iquitos city of Peru in weeks.

The fact that they are perfectly correlated with each other (value 1) is a clear sign that they are. This says that mosquitoes live in areas with high humidity. Since temperature plays a vital role in the spread of mosquitoes, it is correlated both with each other and with the total number of cases. Surprisingly, the weakest part of the year is also highly correlated to San Juan City, and as a result, we will be keeping a close eye on that. In addition, if we plot “a number of years” against “week of the year,” we find that there is an outbreak at the end of the year in both cities. We arrived at this conclusion after outlining the plot between the two variables. The number of reported cases grows, and outbreaks often occur over a few weeks, as illustrated in Table 5 and Figure 8 and Figure 9, respectively.

## 4. Results of Different Classifiers

In this instance, we are using a variety of machine learning classifiers, beginning with KNN and moving on to decision trees, random forests, Gaussian neighbor boundaries, and support vector classifiers. In this instance, we are utilizing k-fold cross-validation to partition the data into ten equal portions for the purpose of classification. As a direct consequence of this, the mean value obtained after ten iterations is shown in Table 6.

**Figure 10:**
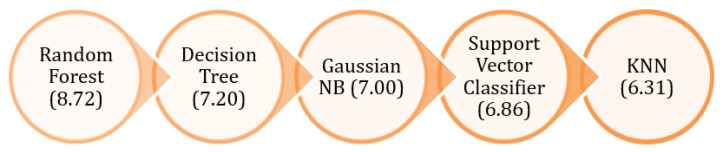
Machine learning classifier ranking based on mean achieved after applying 10-K fold.

It can be seen rather plainly from Table 6 that the random forest classifier is the one that turns out to be the best one, with a mean score of 8.72. For a more transparent illustration of the ranking of classifiers (see Figure 10).

## 5. Discussion and Conclusions

As a result of its popularity and widespread application in image segmentation, deep learning has developed into a crucial instrument and is able to achieve ever-higher levels of precision. However, the primary concern is centered on the optimization of deep learning, and optimization encompasses multiple levels. Some of these levels include perfecting the deep network architectures and carrying out ensembled learning; hyperparameter tuning, which is an empirical method; optimizing the loss function in accordance with evaluation metrics; and making use of the appropriate optimizer and activation functions.

The purpose of this research is to develop a diagnostic model for the disease dengue by using machine learning techniques such as KNN, D.T., R.B., SVR, and GNB. The model will be able to make correct predictions regarding the progression of the disease as well as allow for early diagnosis of the disease. As a result of these upcoming initiatives, the focus of prioritization should be placed on cause–effect models for the diagnosis of disease. Not only is it vital to diagnose the sickness, but it is also essential to analyze the elements that have the most considerable influence on the infection. It is essential to do both things in order to be successful. A more profound comprehension of the etiology of the disease, along with the creation of more correct diagnostic models, would be of tremendous assistance in the fight against dengue fever, as well as in the reduction of complications and fatalities caused by the disease. The use of modeling for the purpose of minimizing the impact of data uncertainty is another vital area. One of the primary challenges that must be surmounted before the quality of previously developed models can be enhanced is the poor standard of epidemiological data about dengue. As a last consideration, the use of independent loops of data analysis works to automate the decision-making process in disease control. Although the D.T., KNN, SVR, and GNB methods all generate better results, the R.F. method requires significantly more time to compute since it generates superior results. Based on the findings, it appears that the R.F. technique is the one to choose. Because of this, it has been determined that, out of all these various machine learning algorithms, the RF-based diagnostic model is the one that is best suited for accurately diagnosing dengue fever at an earlier stage. This conclusion was reached because of the reasons.

The substantial number of optimization factors and schemes that needed to be conducted empirically in order to give our final design requirements were the primary obstacles that needed to be overcome in this effort. Even if we have scaled back the trainable parameters of the network such that they are more compatible with the hardware, there is still the issue of the significant amount of CPU power that must be present to complete the training.

In conclusion, we can say that reason-based models can help with the analysis and interpretation of dengue disease data. This is something that we can assert. Because there is a severe lack of high-quality data in the field of healthcare, machine learning models that can deal with ambiguity can be highly valuable. In conclusion, data decentralization, in conjunction with aggregated learning, may make it possible to cut the costs of computer modeling and may also make it possible to do so without compromising the data’s integrity. This may be possible.

## Figures and Tables

**Table 1:** A literature review regarding the dengue fever forecast.

Year	Reference	Machine Learning/Other Techniques	Findings
1995	[37]	CIMSiM (Container-Inhabiting Mosquito Simulation Model) DENSiM (Dengue Simulation Model)	Specifics regarding the weather Bring the model to a close with an example (entomologic, demographic, and epidemiologic)
2001	[38]	CIMSiM (Container-Inhabiting Mosquito Simulation Model)	Rates of development and survival, global-scale and long-term climate data, monthly mean temperature, precipitation, and cloud cover
2002	[39]	Mathematical Approach	Recruitment rate Biting rate Vector mortality The 2-serotype model is considered
2004	[40]	ELISA Method, which stands for “Enzyme-Linked ImmunoSorbent Assay.” RNA (RiboNucleic Acid) Extraction	Hemagglutination inhibition Complement fixation The test of neutralization MAC-ELISA (IgM antibody capture enzyme-linked immunosorbent assay) (IgM antibody capture enzyme-linked immunosorbent assay) Indirect immunoglobulin G ELISA
2005	[41]	ANN MATLAB Toolbox	Input neurons equal 9, hidden neurons equal 5, and output neurons equal 1. Iterations equal 25, accuracy equals 90%, and error equals 10%. Clinical and epidemiological data: total patients 252: (4 DF and 248 DHF patients). Accuracy in predictions of 90% An inaccuracy in the forecasting of 10%
2007	[42]	ELISA Method	(IP-10 and I-TAC, A549, MG-132 and ALLN) New host genes associated with dengue sickness have been identified through objective research on gene expression, and these genes have been validated through available research.
2008	[43]	Wavelet, SVM, SVR, GA	Rainfall, humidity, temperature
2008	[44]	Decision Tree (C4.5)	Complete blood count, white blood cell/lymphocyte count The temperature of the body, hemoglobin count, or both Neutrophil count The total number of patients is 1200, of which 364 have dengue and 836 do not. The overall error rate is equal to 15.7% (after k-fold validation)
2008	[45]	PCR (Polymerase Chain Reaction)	Hematological and biochemical Total patients 104: grade I DHF = 66; grade II DHF = 34; grade III DHF = 4; grade IV DHF = 3
2008	[46]	SARIMA (Seasonal Autoregressive) Integrated Moving Average) Models	Dataset from January 2000 to September 2007
2008	[47]	SPSS mRNA (messenger RNA) Analysis	(NFKB1, NFKB2, TNFR1, IL1B, IL8, and TNFA) (TLR7, TLR4R3, TLR1, TLR2, TLR4R4, and TLR4 co-factor CD14) DHF = 56 children
2009	[48]	Rough Set Theory	Headache Vomiting Temperature
2010	[22]	SVM RBF	The number of genes is 12, including MYD88, TLR3/7/9, RIG1, IRF3/7, CLEC5A, IFN-/, and MDA5. The MYD88 and TLR7 RBF kernel functions, which have a c value of 1.0 and a c value of 10, respectively, are the most efficient genes. Patients = 28; 15 DF, 13 DHF
2010	[49]	ANN	Mean temperature. Relative humidity of the air total rainfall Total dataset = 14,209 (dengue confirm cases)
2010	[43]	PDB (Protein Data Bank) codes cryoEM (Cryo-electron Microscopy)	E glycoprotein (envelope protein) M protein (membrane protein)
2010	[4]	SVM RBF	Structural proteins consist of three components: capsid (C), membrane (M), and envelope (E). The number of non-structural proteins equals 7 (NS1, NS2A, NS2B, NS3, NS4A, NS4B, and N55)
2010	[50]	Rough Set Analysis	Temperature Headache Vomiting
2010	[19]	ANN	Temperature Rainfall Relative humidity Result: highest accuracy 85.92%
2011	[51]	Regression in a linear fashion (Step-down) Generalize Boosted regression. negative binomial Regression Logistic regression SVM	The SVM Model achieved a better result in the logistic regression (in both locations): Area under curve (AUC) for the SVM models with the use of a cutoff of 0.906 (in Singapore) and 0.960 for the 75th percentile (in Bangkok)
2011	[52]	Spatio-temporal Analysis	There are four criteria to consider: volume, location, time, and content. The cluster approach can be used on Twitter to anticipate dengue fever outbreaks locally and in the immediate future.
2012	[53]	CART Method Random Forest Method	Carry out ten separate experiments, each with tenfold independent validation. The overall accuracy rate is an average of 84.0% (for D.F.) and 84.6% (for DHF) AUC = 0.87

**Table 2:** Data in a separate dataset.

Dataset	Data
Training	1456
Testing	416
Total	1872

**Table 3:** Statistical characteristics along with their respective correlation values (1 as perfectly correlated and 0.9 as almost perfectly correlated).

Parameter	Correlation (1/0.9)
** reanalysis_sat_precip_amt_mm and precipitation_amt_mm **	1
** reanalysis_specific_humidity_g_per_kg and reanalysis_dew_point_temp_k **	1
** ndvi_nw **	0.9
** ndvi_ne **	0.9
** reanalysis_avg_temp_k **	0.9
** reanalysis_air_temp_k **	0.9
** reanalysis_tdtr_k **	0.9
** reanalysis_max_air_temp_k **	0.9
** station_diur_temp_rng_c **	0.9
** reanalysis_tdtr_k **	0.9

**Table 4:** Selected features for different cities.

City	Features	Labels
San Juan	936, 24	936, 4
Iquitos	520, 24	520, 4
Total Features	1456, 24	1456, 4

**Table 5:** Increase in outbreaks and cases in two cities (San Juan/Iquitos) in weeks.

City	Increase in Cases (Range in Weeks)	Increase in Outbreak (Range in Weeks)
San Juan	35th–45th	35th–45th
Iquitos	45th–50th	45th–50th

**Table 6:** Analysis result of different machine learning classifiers to classify dengue disease by using 10-K-fold cross-validation and mean as a result of 10 iterations.

ML Classifier	K Fold = 10										Mean
	Scoring Accuracies										
	1	2	3	4	5	6	7	8	9	10	
KNN	0.095890	0.034246	0.082191	0.082191	0.082191	0.054794	0.041379	0.027586	0.062068	0.068965	6.315068
Decision Tree	0.075342	0.068493	0.109589	0.068493	0.068493	0.109589	0.055172	0.068965	0.048275	0.048275	7.206896
Random Forest	0.075342	0.082191	0.082191	0.143835	0.047945	0.109589	0.075862	0.096551	0.082758	0.075862	8.721303
Gaussian NB	0.075342	0.082191	0.047945	0.068493	0.047945	0.095890	0.062068	0.048275	0.089655	0.082758	7.005668
Support Vector Classifier	0.068493	0.068493	0.061643	0.068493	0.061643	0.075342	0.062068	0.055172	0.075862	0.089655	6.868682

## Data Availability

Not applicable.
